# Cardiotoxicity in Breast Cancer: Impact of Clinical Classifications and Treatment on Heart Health

**DOI:** 10.3390/cancers16244281

**Published:** 2024-12-23

**Authors:** Sergiu Ioan Murg, Loredana Matiș, Andrada Florina Moldovan, Andrada Florina Schwarz-Madar, Daniela Florina Trifan, Timea Claudia Ghitea, Mircea Ioachim Popescu

**Affiliations:** 1 Doctoral School, Faculty of Medicine and Pharmacy, University of Oradea, 410068 Oradea, Romania; sergiumurg@yahoo.com (S.I.M.); schwarz_andrada@yahoo.com (A.F.S.-M.); 2 Department of Clinical Discipline, Faculty of Medicine and Pharmacy, University of Oradea, 410068 Oradea, Romania; matisloredana@yahoo.com (L.M.); onita.andrada@yahoo.com (A.F.M.); procardia_oradea@yahoo.com (M.I.P.); 3 Pharmacy Department, Faculty of Medicine and Pharmacy, University of Oradea, 410068 Oradea, Romania

**Keywords:** cardiotoxicity, cancer therapies, chemotherapy-induced heart failure, HER2-targeted therapies, left ventricular ejection fraction (LVEF)

## Abstract

Background/Objectives: Cardio-oncology has become essential in addressing cardiovascular complications from cancer therapies. While advancements in treatments have improved survival rates, they also increase cardiovascular risks. This study evaluates the cardiotoxic effects of cytostatic treatments, examining the relationship between tumor characteristics, such as histopathology and TNM classification, and cardiovascular complications, aiming to improve cardiotoxicity prevention and management in oncology patients. Methods: We conducted a retrospective analysis of cancer patients treated with anthracyclines, HER2-targeted therapies, and radiotherapy. Cardiac function was monitored via echocardiography, focusing on global longitudinal strain and left ventricular ejection fraction (LVEF). Cardiac troponins and natriuretic peptides were measured to detect subclinical cardiotoxicity, with patients stratified by TNM cancer stage and histopathology. Results: Our analysis identified a significant association between certain cytostatic treatments, such as anthracyclines and HER2-targeted therapies, and a reduction in LVEF, particularly in patients with advanced-stage cancer. Biomarker assessments indicated early signs of cardiotoxicity in patients before clinical symptoms emerged. The findings also demonstrated a higher prevalence of cardiovascular complications in patients with pre-existing risk factors. Conclusions: This study highlights the importance of personalized treatment protocols in minimizing cardiotoxicity and improving the quality of life for oncology patients. Regular cardiac monitoring, combined with the use of biomarkers, can help identify high-risk patients early, allowing for timely interventions. Future research should focus on optimizing cardioprotective strategies to mitigate the cardiovascular risks associated with modern cancer therapies. Clinical Trial Registration: N/A (retrospective study).

## Simple Summary

Cancer treatments, while improving survival rates, can have serious side effects on the heart. This study looks at how certain cancer therapies, such as chemotherapy and targeted treatments, can cause heart problems. By closely monitoring patients’ heart function and using specific biomarkers to detect early signs of heart damage, we aim to identify which patients are most at risk and when these problems might occur. Our goal is to improve the way cancer patients are treated by finding better ways to protect their heart health during treatment. The findings from this research could help doctors develop more personalized approaches to cancer care, ensuring that patients receive effective cancer therapies while minimizing the risk of long-term heart complications.

## 1. Introduction

Cancer survivors are exposed to various risks, including cancer recurrence and cardiovascular events. Cardio-oncology promotes a multidisciplinary approach throughout the treatment process, ensuring efficient management of oncological patients with cardiovascular complications [1]. However, research in this field is still in its infancy, with some limitations. The positive results obtained in many studies may vary depending on age, sex, race, marital status, cancer stage, time of diagnosis, and surgery. Therefore, extensive research is needed to confirm these findings [2]. Additionally, many aspects regarding the interactions between cardiovascular and antitumor drugs, as well as the influence of genes on the mechanisms of these treatments, remain unexplored. Clinical trials are needed to determine the optimal time to interrupt or resume oncological therapy due to cardiovascular complications and to identify alternative therapies when necessary [3].

In patients with both cardiovascular disease and cancer, physicians must adopt appropriate treatment strategies to minimize the adverse effects of antitumor therapy on the cardiovascular system. Oncologists should be well-informed about the risks of cardiovascular complications, such as heart failure, coronary heart disease, hypertension, valvular heart disease, and arrhythmias, to effectively prevent, diagnose, and treat these side effects. Cardiologists, in turn, must be aware of the increased tumor risk associated with certain cardiovascular drugs in vulnerable patients [4,5].

Given the risk of cardiotoxicity, especially with innovative oncological therapies, there is a clear need for updated therapeutic algorithms based on the latest research and clinical guidelines. Cardio-oncology, through a multidisciplinary approach, offers integrated care for patients with cancer and cardiovascular diseases. This discipline plays a crucial role in optimizing care by preventing, diagnosing, and treating cardiovascular complications associated with oncological therapies. The proposed study analyzes the cardiotoxic effects of anticancer treatments and presents new diagnostic and therapeutic approaches to improve outcomes for these patients [6,7].

Over the years, oncology and cardiology have become strongly integrated. Cardiologists specialized in cardio-oncology are essential in collaborating with oncologists, which will lead to advancements in both disciplines. Cardio-Oncology Rehabilitation and Education (CORE), inspired by the Cardiac Rehabilitation (CR) model, holds promise for cancer survivors [8]. Health policymakers should expand CR programs to include cancer patients, aiming to reduce mortality. Cardio-oncology must expand beyond the treatment of cardiotoxicity, and oncologists must adopt effective tools for the prevention and treatment of cardiovascular disease (CVD), including drugs and lifestyle changes such as structured exercise [9].

Clinical studies have shown that cancer is a significant contributor to noncardiac mortality among patients with heart failure (HF), and these patients are at increased risk of developing cancer [10]. This link may be partly explained by common risk factors, such as aging, obesity, and smoking. However, growing experimental evidence suggesting a causal relationship between HF and cancer necessitates a reevaluation of the connection between these two diseases by the scientific and medical communities. Both HF and cancer are associated with systemic manifestations, and the interaction between them is becoming increasingly evident. This phenomenon has been termed “reverse cardio-oncology” [11].

One of the major challenges in breast cancer treatment is cardiotoxicity induced by allopathic therapies, particularly HER2-targeted therapies and chemotherapy. Trastuzumab and other HER2-targeted monoclonal antibodies are effective in treating HER2-positive breast cancer but can cause cardiotoxicity, especially congestive heart failure (CHF) [12]. Trastuzumab combined with anthracycline chemotherapy increases the risk of heart damage. Studies have shown that 8.7% of patients treated with trastuzumab experience significant decreases in left ventricular ejection fraction (LVEF), potentially leading to heart failure [13,14].

Pertuzumab, another antibody used in the treatment of HER2-positive breast cancer, has a similar cardiotoxicity profile, albeit to a lesser extent. The combination of pertuzumab and trastuzumab may exacerbate cardiac effects, though the clinical benefits remain significant for patients [15]. Antibody-drug conjugates, such as trastuzumab-emtansine (T-DM1), have demonstrated efficacy in treating breast cancer with reduced cardiotoxicity compared to standard trastuzumab. Recent studies show that trastuzumab-deruxtecan (T-DXd) has significantly less cardiotoxicity but carries other risks, such as interstitial lung disease [16].

This manuscript aims to evaluate the cardiotoxic effects of cytostatic treatments according to the clinical stage of cancer, histopathological examination, and TNM (Tumor, Lymph Node, Metastasis) classification. The goal is to identify correlations between specific tumor characteristics and the degree of cardiotoxicity, providing in-depth insight into the cardiovascular risks associated with various types and stages of cancer treated with cytostatics. The purpose of this work is to provide concrete data that will contribute to the optimization of therapeutic protocols through a personalized approach to cytostatic treatments, considering the histopathological profile of the tumor and the TNM stage. In this way, the prevention and more efficient management of cardiotoxicity can be achieved, ultimately improving the quality of life for oncology patients.

## 2. Materials and Methods

### 2.1. Study Design

This study was designed as a retrospective observational trial aimed at evaluating the impact of breast cancer treatment on cardiotoxicity and its subsequent effects on patients’ quality of life (QOL). The research was conducted at Hospital Oncohelp, Arad, Romania, between January 2008 and December 2023. Ethical approval was obtained from the Ethics Committee of Hospital Oncohelp, Arad, Romania, under approval code CEFMF 8/26 June 2024, and all participants provided written informed consent.

This study adheres to the ethical standards outlined in the Declaration of Helsinki and follows the CONSORT guidelines for observational studies.

Our study focused on chemotherapy and targeted therapies due to their direct cardiotoxicity. While surgery and radiation therapy are essential in breast cancer treatment, their cardiotoxic risks are less direct or long-term and were beyond our study’s scope.

### 2.2. Patient Population

A total of 5149 female patients with histologically confirmed breast cancer, in the age range of 20–88 years old, were enrolled. At the end of the research period, 3706 patients (71.97% across all cancer stages) remained and were included in the final analysis. Patients were selected based on the following inclusion criteria:

Diagnosis of stages I–IV breast cancer.

Patients receiving any standard chemotherapy or HER2-targeted therapies were included to enable the assessment of a diverse population undergoing various treatment regimens.

The study cohort received a variety of chemotherapy regimens, primarily involving HER2-targeted therapies and cytotoxic agents. The most frequently administered agent was Trastuzumab (Herceptin), either alone or in combination with other therapies. In some cases, Trastuzumab was combined with Bondronat (Ibandronic acid) and Tamoxifen to enhance therapeutic efficacy. Another common regimen involved Kadcyla (Trastuzumab Emtansine), administered either as a single agent or in combination with bisphosphonates, such as Acid Ibandronic, for patients who required additional bone support.

Other regimens included Paclitaxel (TAX) combined with Trastuzumab and sequential administration of Docetaxel and Cyclophosphamide alongside Trastuzumab for patients with advanced disease. The use of Trastuzumab Emtasinum was noted in some cases, typically administered on a 21-day cycle, with dosages occasionally adjusted to 80% based on patient tolerance. These various regimens reflect the personalized approach taken to optimize treatment efficacy while managing cardiotoxicity risks associated with HER2-targeted therapies and chemotherapy.

Adequate organ function and ability to complete the study questionnaires.

The exclusion criteria were:

Patients were excluded if they had a history of significant cardiovascular disease (based on European Society of Cardiology (ESC) guidelines), including diagnosed coronary artery disease, congestive heart failure, or a prior myocardial infarction. Additional exclusion criteria included any pre-existing conditions that would impair the ability to complete study assessments or those unable to provide informed consent.

### 2.3. Breast Cancer Treatment and Cardiotoxicity Monitoring

Patients received treatment based on established clinical protocols. Cardiotoxicity was monitored as part of the study due to the known effects of certain chemotherapy agents (e.g., anthracyclines, trastuzumab) on cardiac function. Cardiac monitoring involved regular assessments of left ventricular ejection fraction (LVEF) using echocardiography at baseline, midway through treatment, and at completion of therapy. Cardiotoxicity was defined according to the Common Terminology Criteria for Adverse Events (CTCAE) version 26, as a reduction in LVEF by more than 10% (≥10%) from baseline to a value < 50%.

### 2.4. Quality of Life Assessment

The primary outcome, quality of life, was measured using the validated EORTC QLQ-C30 (European Organization for Research and Treatment of Cancer Quality of Life Questionnaire—Core 30) questionnaire in conjunction with the breast cancer-specific module QLQ-BR23 [17]. The EORTC QLQ-C30 assesses five functional scales (physical, role, emotional, cognitive, and social functioning), three symptom scales (fatigue, nausea/vomiting, pain), and a global health status scale. Administration of Questionnaires: Patients completed the questionnaires at three key points:

Baseline: before initiation of cancer treatment.

Mid-treatment: after 3 cycles of chemotherapy.

End of Treatment: within 6–8 weeks following the last dose of treatment.

The questionnaires were administered electronically via a secure platform or in paper format, depending on patient preference. Assistance was provided as needed, ensuring all responses were accurately recorded.

Scoring and Interpretation

All responses were scored according to the EORTC scoring manual. Scores were transformed into a 0–100 scale:

For functional scales and global health status, higher scores indicated better functioning and quality of life.

For symptom scales, higher scores indicated greater symptom severity or more significant issues.

### 2.5. Statistical Analysis

Data were analyzed using SPSS, version 20 [18]. Descriptive statistics were used to summarize demographic and clinical characteristics. Changes in QOL scores over time were analyzed using tests such as paired *t*-tests, ANOVA, or Wilcoxon signed-rank tests, depending on data distribution. The correlation between cardiotoxicity and QOL scores was assessed using Pearson correlation coefficients.

To account for missing data, a complete case analysis was conducted. Statistical significance was defined as *p* < 0.05.

### 2.6. Data Availability

All relevant data used in this study, including de-identified patient data and raw scores, have been deposited in the publicly available repository. Any computer code used for analysis is available upon request, and all materials used in this study will be made available to readers upon reasonable request.

### 2.7. Ethical Considerations

This study was conducted in accordance with the ethical guidelines of the Declaration of Helsinki. Approval for human subject research was granted by the University of Oradea, Ethics Committee, approval code no. CEFMF 8/26 June 2024. Informed consent was obtained from all participants, and all data were anonymized prior to analysis to ensure patient confidentiality.

### 2.8. Protocols and Materials

All protocols used for data collection, cardiotoxicity evaluation, and QOL assessment follow established standards. The use of the EORTC QLQ-C30 and QLQ-BR23 is well-established and appropriately cited. Any new methodologies developed for this study, such as a special evaluation of the period of allopathic treatments, to follow the quality of life from the perspective of the cardiotoxicity of the treatment.

## 3. Results

### 3.1. Demographic Description

The cohort comprised 5149 patients with an age range of 20 to 88 years (mean age = 57.48, SD = 12.51). The mean weight was 70.14 kg (SD = 24.30), ranging from 37 to 96.5 kg, and the mean height was 161.66 cm (SD = 7.51), with a range from 140 to 184 cm. Among the participants, 39.7% had comorbidities, including hypertension, diabetes, chronic liver diseases, chronic kidney diseases, and chronic gastrointestinal diseases, with no history of malignancies in any patients. Treatment types included standard chemotherapy and HER2-targeted therapies, allowing for an evaluation of diverse therapeutic regimens within this patient population.

The study included 7674 patients (Table 1), of whom 32.91% (2525 individuals with a mean age of 61.31 years, SD = 14.87) had significant cardiovascular diseases and were excluded. The focus of the study was on evaluating the cardiotoxic effects of drug treatments. This resulted in a final cohort of 5149 individuals with a mean age of 57.48 years (SD = 12.51).

**Figure 1:**
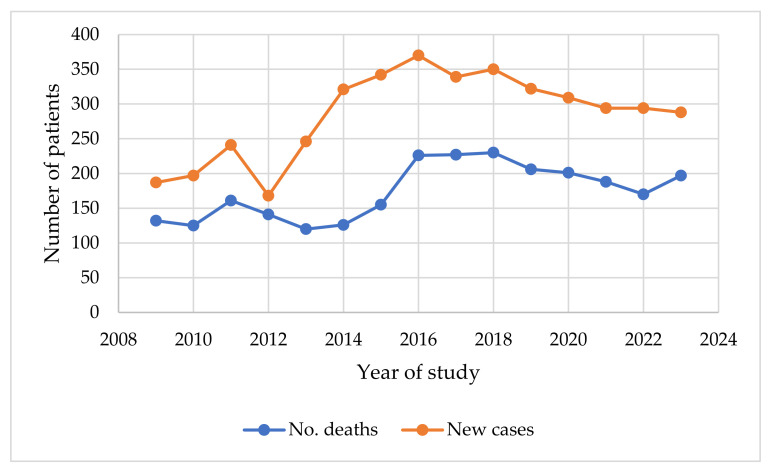
Trend in number of deaths and new cases from 2009 to 2023.

Figure 1 shows an upward trend in new cases from 2009 to 2016, peaking at 370 and then stabilizing with slight fluctuations, ending at 288 in 2023. Deaths also fluctuated, peaking in 2018 at 230 but gradually declining to 170 in 2022 before a small rise to 197 in 2023. The increasing cases with relatively stable or decreasing deaths suggest potential improvements in treatment, early detection, or patient management, reflecting better survival rates over time.

**Figure 2:**
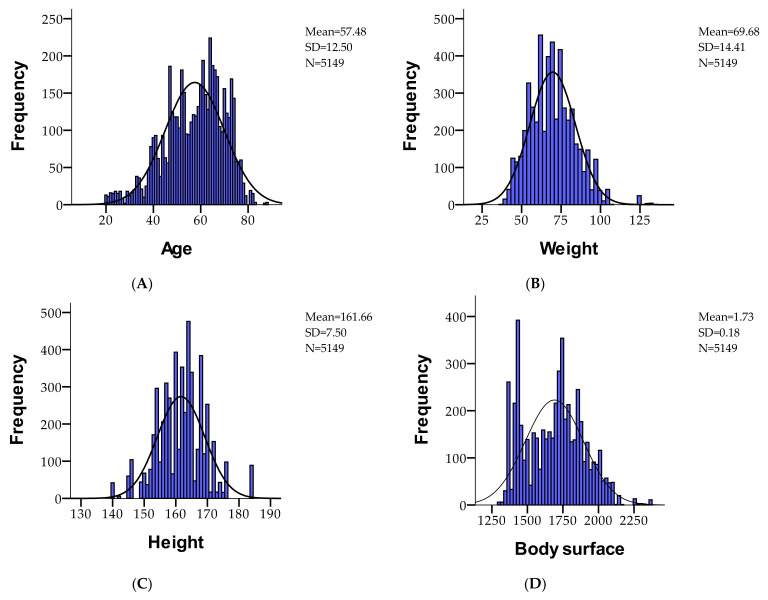
Demographic description of the study cohort regarding age (**A**), weight in kg (**B**), height in cm (**C**), and body surface in kg/m^2^ (**D**).

Table 2 provided descriptive statistics for several variables, including LVEF (a respiratory function metric), heart problems, age, weight, height, body surface area, and a performance index, based on a dataset of 5149 valid cases with no missing data. The mean LVEF is 0.9598, with a standard deviation of 0.19645, indicating a strong left-skew (−4.683) and a high kurtosis (19.937). Heart problems occur in about half the cases (mean = 0.5053), and the participants have an average age of 57.48 years (SD = 12.51). The average weight is 69.68 kg (SD = 14.41), height is 161.66 cm (SD = 7.51), and body surface area is 1.73 m^2^ (SD = 0.18). The performance index has a mean of 0.6059, with a relatively low skew (−0.085) and kurtosis of −1.225, indicating some variability in these measures. The ranges for age, weight, height, and body surface area show significant variation, and skewness and kurtosis highlight the distributional characteristics of each variable. The demographic description of the study cohort regarding age, weight in kg, height in cm, and body surface in kg/m^2^ is presented in Figure 2.

This study shows the frequency distribution of LVEF values in a sample of 5149 individuals. The majority (96%) have LVEF values between 50% and 65%, while 4% have LVEF values below 50%. The cumulative percentage confirms that 100% of the sample falls within these two categories. Relating to the distribution of heart problems among 5149 individuals, 50.5% of the sample reported having heart problems, while 49.5% did not. The majority (57.3%) have a performance index of 1.00, while 41% have a performance index of 0.00. A small proportion (1.6%) have a performance index of 2.00.

### 3.2. Differential Diagnosis

**Figure 3:**
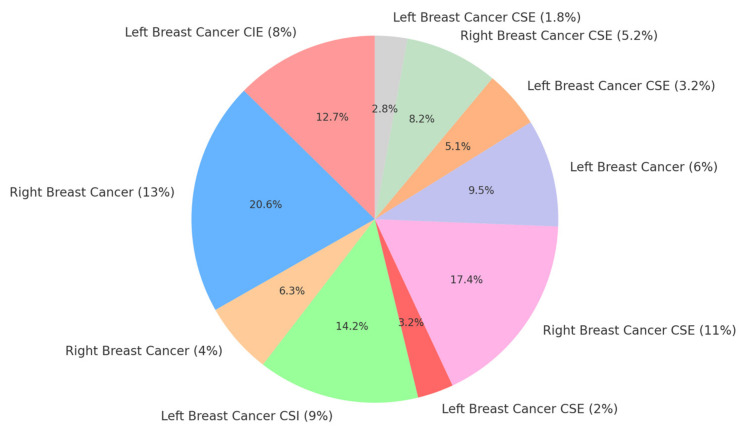
Distribution of breast cancer cases by side and classification, where CSE = Clinical Staging Examination, CSI = Cancer Staging Investigation, and CIE = Clinical Imaging Evaluation.

In our study, we encountered a variety of breast neoplasm cases, each with specific characteristics and treatments. One case involved a mammary neoplasm in the left breast, where the patient underwent a modified radical mastectomy and left axillary lymphadenectomy in October 2014, followed by chemotherapy with four courses of AC between November 2014 and January 2015. The patient was also diagnosed with hypertension and type 2 diabetes and had previously undergone a total thyroidectomy for a multinodular goiter in October 2013. This case represented 8% of the total cases. The distribution of Breast Cancer Cases by Side and Classification is presented in Figure 3.

Another patient was diagnosed with a breast neoplasm in the right breast, treated with six courses of Cyclophosphamide, Methotrexate, and Fluorouracil (CMF) and Herceptin between June and November 2014, followed by a mastectomy and removal of the right axillary nodes in December 2014. This case represented 13% of the total number of cases.

In another instance, a patient with a mammary neoplasm in the right breast and carcinomatous mastitis underwent a biopsy and received chemotherapy with six courses of ET between November 2013 and March 2014. The patient also received irradiation with a total dose of 50 Gy between May and June 2014. Additionally, the patient had grade II essential hypertension, chronic ischemic heart disease, and grade II obesity. This case represented 4% of the total cases.

Another case involved a mammary neoplasm in the left breast, treated with a Madden mastectomy and axillary lymphadenectomy in August 2013. The patient also had rheumatoid arthritis. This case represented 9% of the total cases.

A patient diagnosed with a mammary neoplasm in the left breast underwent a sectorectomy and left axillary lymphadenectomy on 28 October 2014, while also being treated for essential hypertension and type 2 diabetes. This case constituted 2% of the total cases.

In another case, a left breast neoplasm was treated with chemotherapy (four courses of Epirubicin and Cyclophosphamide (EC), four courses of Docetaxel, and Herceptin between February and July 2014), along with irradiation of 50 Gy to the left breast and 50 Gy to the left axillary and supraclavicular nodes in August and September 2014. This case represented 11% of the total cases.

Another patient presented with advanced left breast neoplasm with pleural, lymph node, and liver metastases, representing 6% of the cases.

A patient with a left breast neoplasm underwent a left radical mastectomy and received chemotherapy with six courses of EC. The patient is currently undergoing hormone therapy and also had a left femoral neck dislocation and mild secondary anemia. This case constituted 2.3% of the total cases.

Another case of left breast neoplasm was complicated by multiple bone (vertebral, costal, scapular), bilateral pulmonary, and supraclavicular lymph node metastases. The patient underwent radical mastectomy and left axillary lymphadenectomy in July 2012 and received chemotherapy with eight courses of EC between August 2012 and January 2013. This case represented 5.2% of all cases.

Finally, a patient with left breast neoplasm was treated with chemotherapy (one course of EC in June 2014), followed by a sectorial mastectomy in July 2014 and additional treatment with six courses of Docetaxel and Herceptin between August and December 2014. The patient was irradiated with total doses of 48 Gy and 50 Gy to different areas of the left breast and axillary nodes in February 2015. This case constituted 1.8% of the total cases.

### 3.3. Histopathology

The histopathological analysis identified several characteristics of infiltrative ductal carcinoma and other forms of breast cancer, revealing significant variations in tumor typology and aggressiveness.

In the first case, infiltrative ductal carcinoma was diagnosed, presenting with perineural invasion and lymphatic emboli. The immunohistochemical (IHC) profile of the tumor indicated estrogen receptor (ER) expression at 95%, progesterone receptor (PR) expression at 98%, a Ki67 index of 30%, and a HER2/neu score of +3. This suggested a tumor with increased proliferative potential and a favorable response to hormonal treatments but with possible increased aggressiveness due to HER2 positivity.

In January 2012, a cytological examination confirmed the presence of infiltrative ductal carcinoma. By July 2012, histopathology revealed residual invasive ductal carcinoma with perineural invasion and involvement of 6 of 12 lymph nodes. The IHC profile showed an ER expression of 20%, a PR expression of 90%, and a Ki67 index of 30%. HER2 was assessed as 2+, and FISH testing was pending to assess gene amplification.

In January 2013, a grade G3 invasive ductal carcinoma involving 3 of 13 lymph nodes was identified. The IHC profile showed a HER2 index of 3+, a Ki67 of 90%, and 0% expression of both ER and PR. In September 2013, lymph nodal metastases were confirmed, with a biopsy of the supraclavicular nodes showing negative ER and PR, a Ki67 index of 30%, and HER2 of 3+.

Another case involved G2 grade invasive ductal carcinoma with no lymphatic or vascular invasion and involvement of 2 of 17 lymph nodes. The IHC profile showed ER expression of 90%, PR of 0%, a Ki67 of 10%, and HER2 of 2+, with gene amplification confirmed by CISH.

Other cases included invasive ductal carcinoma with a trabecular, glandular, and solid growth pattern associated with invasive lobular carcinoma. The IHC profile showed ER of 90%, PR of 90%, a Ki67 of 30%, and HER2 of 2+. In October 2013, a biopsy revealed carcinomatous emboli in the lymphatic vessels, with ER and PR expression at 0%, unspecified Ki67, and HER2 of 2+, with positive gene amplification.

Additional diagnoses included high-grade, poorly differentiated ductal carcinoma with ER expression of 90% and a Ki67 index of 80%. Another case was classified as G3 invasive ductal carcinoma, involving one of nine lymph nodes, with an ER profile of 60%, PR of 75%, and HER2 of 2+, with gene amplification confirmed by CISH. Thus, the histopathological analysis showed a varied spectrum of breast tumors with significant differences in immunohistochemical profiles and degrees of aggressiveness, suggesting that the management of each tumor must be personalized according to the individual characteristics of each case (Table 3).

### 3.4. TNM

The TNM classification (Table 4) is an essential system in oncology for assessing the stage of cancer, based on three main components: T for the size and extent of the primary tumor, N for regional lymph node involvement, and M for the presence of distant metastases. This detailed TNM classification helps determine how advanced the cancer is and aids in planning the appropriate treatment strategy for each patient. In the context of the data presented, here is an explanatory description of the various TNM stages:

Advanced stages with extensive involvement:

pT4b Nx M1 (hep, lung, skin): The primary tumor is advanced (T4b), indicating significant local extension, possibly invading adjacent structures. Complete lymph node information is not available (Nx), and metastases are present in the liver, lungs, and skin.

T4c N2 M1 (oss, pul, lym): The primary tumor is T4c, with extensive local extension. Lymph node involvement is N2, indicating significant regional involvement. Metastases are present in the bones, lungs, and lymph nodes.

T4d Nx M1 (oss, hep, pul, lym): The primary tumor is T4d, with extreme local extension. Metastases are present in the bones, liver, lungs, and lymph nodes. Complete information on regional lymph nodes is not available.

Advanced stages with nodal and metastatic involvement:

pT2 N1 M1 (oss, pul, lym): The primary tumor is T2, with moderate size and extension. Lymph node involvement is N1, and metastases are detected in the bones, lungs, and lymph nodes.

pT1 N1 M1 (pul): The primary tumor is small (T1), with involvement of one lymph node (N1) and metastases present exclusively in the lungs (M1).

cT4b N1 M0: The primary tumor is T4b, with advanced local extension and involvement of a lymph node (N1), but no distant metastases (M0).

Intermediate and local stages:

T3 N1 M0, ypT2 N1 M0: The primary tumor is T3, with large size and moderate local extension. Lymph nodes are involved (N1), but no distant metastases are present (M0). After treatment, the stage was reassessed to ypT2 N1 M0, indicating a smaller primary tumor and no distant metastases.

T2 N0 M0: The primary tumor is T2, with moderate size and extension, no lymph node involvement (N0), and no distant metastases (M0).

T1b N1 M0, ypT1c N1a M0: The primary tumor is small (T1b), with involvement of a lymph node (N1), and no distant metastases (M0). After treatment, the stage was re-evaluated as ypT1c N1a M0, indicating a slightly altered primary tumor and no distant metastases.

Stages with limited invasion and no metastases:

pT2 N0 M0: The primary tumor is T2, moderate in size, with no lymph node involvement (N0) and no distant metastases (M0).

T4d Nx M0: The primary tumor is T4d, with extreme local extension but no lymph node information (Nx) and no distant metastases (M0).

pT1c N0 M0: The primary tumor is small (T1c), with no lymph node involvement (N0) and no distant metastases (M0).

Recurrences and metastases:

pT1 N1 M1 (pul): The primary tumor is small (T1), with involvement of a lymph node (N1) and metastases to the lungs (M1).

cT3 N1 M1 (oss), ypT2 N0 Mx: The primary tumor is T3, with moderate local extension and involvement of a lymph node (N1), and bone metastases (M1). After treatment, the stage was reassessed to ypT2 N0 Mx, indicating a smaller primary tumor, but incomplete information on distant metastases (Mx).

### 3.5. Cardiotoxicity

Congestive heart failure is a significant adverse reaction to trastuzumab treatment, characterized by a decrease in left ventricular ejection fraction (LVEF), with an increased risk in elderly patients or those with pre-existing risk factors. However, this decrease is reversible, and guidelines recommend regular monitoring of LVEF and the use of cardioprotective therapies to prevent or manage cardiac dysfunction, without necessarily discontinuing HER2 therapy in patients with mild to moderate dysfunction.

**Figure 4:**
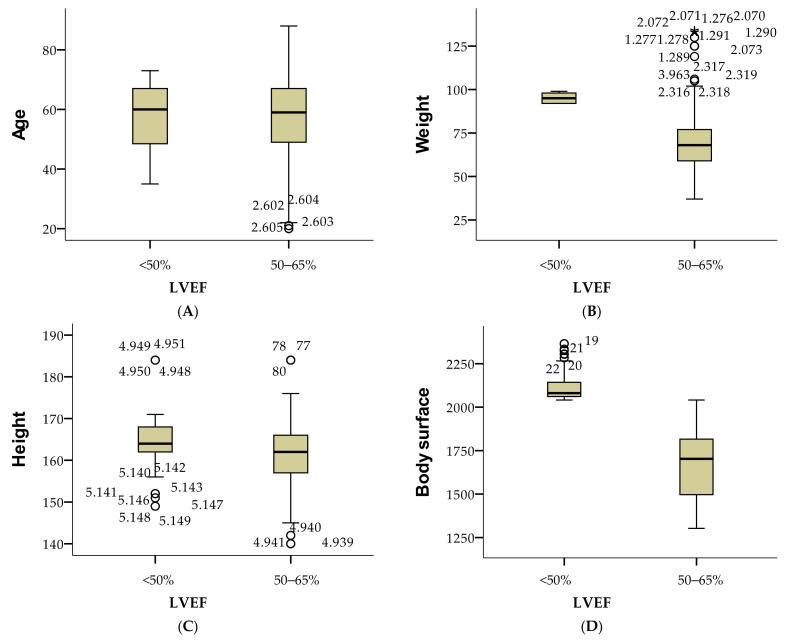
Comparison of health metrics by LVEF categories as age (**A**), weight (**B**), height (**C**), and body surface (**D**).

Figure 4 presents the mean and standard deviation for age, weight, height, body surface area, and performance index across two categories of LVEF: “<50%” and “50–65%”. For individuals with LVEF <50%, the mean age is 57.92 years, weight is 94.99 kg, height is 164.53 cm, body surface area is 2.008 m^2^, and the performance index is 0.63. For those with LVEF between 50 and 65%, the mean age is 57.46 years, weight is 68.62 kg, height is 161.54 cm, body surface area is 1.720 m^2^, and the performance index is 0.61. Data for the “>65%” category is missing.

Table 5 presents Pearson correlation coefficients between several health-related variables, including LVEF, heart problems, age, weight, height, body surface area, and performance index, using data from 5149 individuals. LVEF is negatively correlated with heart problems (−0.153), weight (−0.359), height (−0.078), and body surface area (−0.308), all statistically significant at the 0.01 level. Heart problems show strong positive correlations with weight (0.597) and body surface area (0.562). Age is weakly but significantly correlated with heart problems (0.138) and weight (0.140), while height has a negative correlation with age (−0.205). Body surface area has the strongest correlation with weight (0.938), and performance index has weak but significant correlations with age (0.247) and heart problems (0.096). Most correlations are statistically significant, with only a few exceptions.

### 3.6. Quality of Life

The data presented compares the quality of life scores, measured by the EORTC QLQ-C30, across different categories of left ventricular ejection fraction (LVEF), a marker of heart function, divided into three groups: <50%, 50–65%, and >65%. For patients with an LVEF of less than 50%, the mean EORTC QLQ-C30 score is 63.17 with a standard deviation of 7.80, indicating that reduced heart function may be associated with a slightly lower, but still relatively stable, quality of life. Patients with normal heart function (LVEF 50–65%) have a mean score of 64.28 with a standard deviation of 8.39, showing a similar quality of life to those with lower LVEF, though with slightly greater variability. Data for the group with LVEF greater than 65% is missing, preventing further comparison. Overall, these findings suggest that patients with both reduced and normal heart function report comparable quality of life, although individual experiences vary within each group. The statistical analysis further explores the relationship between left ventricular ejection fraction (LVEF) and quality of life scores, as measured by the EORTC QLQ-C30, using non-parametric tests. A Mann–Whitney U test was conducted to compare the quality of life scores between different LVEF groups. The results yielded a Mann–Whitney U value of 466,556.000 and a Wilcoxon W value of 488,084.000, with a Z-score of −2.147. The Asymptotic Significance (two-tailed) was 0.032, indicating a statistically significant difference in EORTC QLQ-C30 scores between the groups based on LVEF (*p* < 0.05).

**Figure 5:**
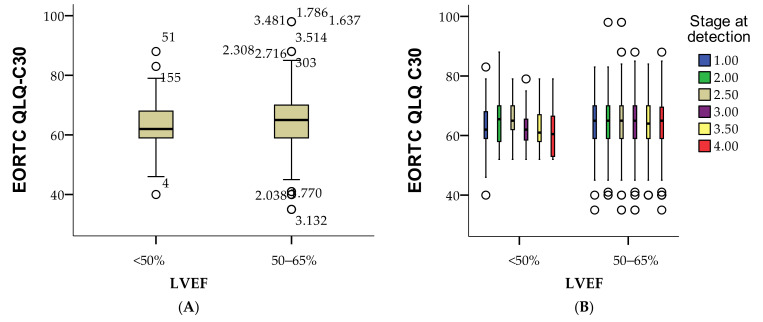
Boxplot presentation of the variation in quality of life in patients according to LVEF per cohort (**A**) and according to cancer stages (**B**), ○ = isolated cases.

This suggests that variations in heart function, as indicated by LVEF, are significantly associated with differences in quality of life. Specifically, patients with lower LVEF (<50%) may experience a slight but statistically significant reduction in their quality of life compared to those with normal or higher LVEF values, reinforcing the importance of monitoring cardiotoxic effects during breast cancer treatment. The grouping variable in this analysis was LVEF, meaning the statistical test specifically examined differences between the predefined categories of LVEF (e.g., <50%, 50–65%), as in Figure 5A. Thus, it can be observed that in stage I, the quality of life is most affected in those with low LVEF, followed by those in stage 3A (Figure 5B).

## 4. Discussion

Recent advances in cancer treatments have significantly improved the survival rates of cancer patients, but they come at a considerable cost to the cardiovascular system [19]. As patient survival has increased, the cardiotoxicity caused by chemotherapy, radiation, and targeted therapies has become a major challenge. Consistent with the findings in our research, multiple studies emphasize the importance of cardiac monitoring during anticancer therapy, particularly with anthracyclines, trastuzumab, and tyrosine kinase inhibitors [20,21].

In line with the literature, anthracyclines are recognized for their potential to induce cardiomyocyte necrosis and fibrosis, a severe dose-limiting cardiotoxic effect [22]. Recent studies show that trastuzumab, a monoclonal antibody, can lead to cardiac arrest, and tyrosine kinase inhibitors can increase blood pressure and impair myocyte contractility [23]. These results reinforce our conclusions regarding the significant impact of these therapies on cardiac function and underscore the need for early identification of high-risk patients.

The lack of significant improvement in outcomes over the years in Romania, as highlighted in Figure 1, underscores the urgent need for enhanced prevention and screening programs to address gaps in early diagnosis and improve survival rates. The 5-year relative survival rate for women with localized breast cancer at the time of diagnosis is about 99%, but this drops significantly to 86% for regional breast cancer and just 31% for distant breast cancer. These figures emphasize the critical importance of early detection in improving prognosis and survival outcomes [24].

Mediastinal or thoracic radiation therapy further exacerbates the risk of cardiotoxicity, contributing to accelerated atherosclerosis, myocardial infarction, and heart failure, highlighting the urgent need for ongoing monitoring [25,26]. In our research, we identified echocardiographic measurements of global longitudinal strain and left ventricular ejection fraction (LVEF) as essential methods for detecting systolic dysfunction. This approach is supported by other studies that suggest 3D echocardiography is a valuable tool for monitoring patients undergoing chemotherapy.

In terms of biomarkers, our review emphasizes the role of cardiac troponins and natriuretic peptides (BNP and NT-proBNP) in the early detection of cardiotoxicity [27,28]. These findings are supported by studies indicating that these biomarkers can signal subclinical cardiac dysfunction before symptoms appear [29]. Ongoing research on myeloperoxidase and microRNAs provides promising new directions for the early detection of cardiac damage [30].

Scientific discussions also stress the need for close collaboration between oncologists and cardiologists, emphasizing the importance of cardiological evaluation before, during, and after oncological treatment [31]. Similarly, the literature recommends customized management plans for patients at high risk of cardiotoxicity to proactively address cardiac dysfunction without compromising the success of cancer treatments [32].

An upward trend in new cancer cases was observed; however, this is counterbalanced by an increasing prevalence of significant cardiovascular diseases among patients, leading to a decrease in the average age of excluded individuals. This highlights the growing impact of cardiovascular risk factors in oncology and underscores the necessity of evaluating chemotherapy-induced cardiotoxicity as a critical component of comprehensive cancer care.

Radiotherapy can cause coronary heart disease and fibrotic changes in the heart valves, pericardium, and myocardium [33,34]. Therefore, patients scheduled to receive chemotherapy, particularly those with a history of cardiovascular disease, should undergo cardiovascular evaluation prior to treatment to minimize the risk of complications. Monitoring left ventricular systolic function and cardiac biomarkers can be beneficial in high-risk populations [35]. Cardioprotective strategies, such as the use of ACE inhibitors, angiotensin receptor blockers, or beta-blockers, can reduce chemotherapy-induced cardiotoxicity. Antiplatelet or anticoagulation therapy may also be considered for patients at risk of hypercoagulability associated with cancer or chemotherapy [36,37].

In an ideal setting, close collaboration between cardiologists and oncologists is essential to manage these adverse effects. Advanced cancer therapy relies on a variety of modalities, including radiation therapy, cytotoxic chemotherapy, targeted molecular inhibitors, and antibodies that modulate the immune response, all of which can negatively impact cardiovascular health [38]. While clinical experience with traditional radiotherapy and chemotherapy is extensive, data on cardiovascular toxicities associated with newer molecularly targeted therapies and immunotherapies remain limited, particularly regarding long-term effects [39]. These late effects are of particular concern, as they have the potential to reduce both the quality of life and prognosis of cancer survivors, especially in those with additional cardiovascular risk factors.

Cardiovascular complications related to cancer treatments include hypertension, venous thromboembolism, coronary heart disease, valvular disease, heart failure, and arrhythmias [40]. Early detection of subclinical cardiotoxicity remains a significant challenge. The multidisciplinary approach offered by cardio-oncology teams is the most effective method for preventing, diagnosing, and treating cardiovascular diseases associated with oncological therapies [41]. This study focuses on the cardiotoxic effects of various cancer treatments and explores new diagnostic and therapeutic methods to improve care for cancer patients. Our results align with Surveillance, Epidemiology, and End Results (SEER) data trends, showing increased breast cancer incidence and decreased mortality, likely due to improved treatments like adjuvant chemotherapy [42]. Similar to SEER’s findings, our data suggest better survival rates, though rising case numbers without reduced advanced cases may indicate overdiagnosis and evolving diagnostic practices.

Elderly patients, those with pre-existing cardiovascular risk factors, and those previously exposed to chemotherapy or radiotherapy are at greater risk of cardiotoxicity [43,44]. Serial evaluations of troponins and the use of echocardiography or cardiovascular magnetic resonance can help detect cardiac involvement early, before the onset of heart failure [45]. Serum biomarkers are becoming increasingly important in the risk stratification and monitoring of patients receiving cardiotoxic therapies, as highlighted in expert panel statements from the European Society of Cardiology [46].

In the context of assessing quality of life in breast cancer patients, the EORTC QLQ-C30 has been widely used as a reliable tool to evaluate the impact of both cancer progression and treatment-related side effects on patient well-being [47,48,49]. Numerous studies in the literature have demonstrated that patients with advanced cancer stages and those experiencing cardiotoxicity, particularly due to treatments like trastuzumab, tend to report lower scores in functional domains such as physical functioning, role functioning, and emotional well-being [12,50]. For instance, previous studies have shown that patients with lower LVEF often report greater fatigue and reduced physical functioning, which aligns with the cardiotoxic effects of treatments. In our study, similar trends were observed, particularly among patients with lower LVEF (LVEF < 50%), who had slightly lower EORTC QLQ-C30 scores compared to those with normal heart function (50–65%). These findings suggest that cardiotoxicity plays a significant role in diminishing quality of life, even in early-stage breast cancer patients. Moreover, while the literature often highlights a greater impact on quality of life in later cancer stages, our results indicate that even patients in stage I with low LVEF experience significant quality of life deterioration, emphasizing the need for early cardiac monitoring and intervention. In relation to quality of life, research has shown that breast cancer patients with sufficient levels of vitamin D often report better physical functioning and less fatigue [51,52,53], which can positively impact their overall EORTC QLQ-C30 scores. Vitamin D deficiency has been linked to worse outcomes, including a higher risk of metastasis and poorer responses to treatments like chemotherapy and trastuzumab. In our study, although vitamin D levels were not directly measured, the potential role of vitamin D in improving quality of life and mitigating some of the cardiotoxic effects of cancer therapies is an area worthy of further investigation. Ensuring optimal vitamin D status may offer a complementary approach to improving both the treatment outcomes and the quality of life in breast cancer patients, particularly those at risk of cardiotoxicity.

Limitations: While our study highlights the importance of cardio-oncology and the need for continuous cardiovascular monitoring during cancer treatment, it is limited by the lack of long-term data on newer molecularly targeted therapies and immunotherapies. Additionally, there remains a need for more research into late-stage cardiovascular effects and their impact on the quality of life in cancer survivors. Despite these limitations, our findings emphasize the critical need for personalized and precise treatment regimens that minimize cardiotoxicity while improving outcomes for oncology patients.

## 5. Conclusions

In conclusion, congestive heart failure is a significant adverse reaction to trastuzumab treatment, primarily characterized by a decrease in left ventricular ejection fraction (LVEF). Elderly patients and those with pre-existing risk factors are at higher risk for developing cardiac dysfunction. However, this decrease in LVEF is generally reversible with proper management. Current guidelines recommend regular monitoring of LVEF and the use of cardioprotective therapies to prevent or manage cardiac issues, allowing for the continuation of HER2 therapy in patients with mild to moderate dysfunction without the need for discontinuation. While this study focused on echocardiographic changes as markers of cardiotoxicity, the integration of biomarkers such as troponins and natriuretic peptides in future research could further enhance early detection strategies. Additionally, our findings highlight that in stage I breast cancer, the quality of life is most affected in those with low LVEF, followed by stage 3A patients. This suggests that both cancer progression and cardiotoxicity have a compounded impact on patient well-being, emphasizing the need for close monitoring of early-stage breast cancer to ensure optimal treatment outcomes and quality of life.

## Figures and Tables

**Table 1:** Demographic and clinical characteristics of the cohort, exclusion group, and study population.

Parameters	Total 7674	Exclusion Group 2525 Persons	Study Group 5149 Persons
Age (±SD)	58.8 (±12.94)	61.48 (±13.48)	57.48 (±12.51)
Weight (±SD)	70.36 (±15.92)	71.77 (±17.79)	69.67 (±14.41)
Height (±SD)	161.18 (±7.12)	160.19 (±6.97)	161.66 (±7.51)
Body surface (±SD)	1.72 (±0.20)	1.71 (±0.21)	1.73 (±0.18)
Performance index (±SD)	0.60 (±0.72)	0.61 (±0.069)	0.60 (±0.075)
Hypertension (%)	1520 (19.80%)	533 (21.11%)	987 (19.17%)
Diabetes (%)	462 (6.02%)	194 (7.68%)	268 (5.21%)
Chronic liver diseases (%)	589 (7.67%)	202 (8.01%)	387 (7.51%)
Chronic kidney diseases (%)	172 (2.24%)	68 (2.68%)	104 (2.02%)
Chronic gastrointestinal diseases (%)	924 (12.04%)	626 (24.78%)	298 (5.79%)

**Table 2:** Descriptive statistics of health and performance variables for a sample of 5149 individuals.

Statistics		LVEF	Heart Problems	Age	Weight	Height	Body Surface	Performance Index
N	Valid	5149						
	Missing	0						
Mean		0.95	0.50	57.48	69.67	161.66	1.73	0.60
Std. Deviation		0.19	0.50	12.50	14.41	7.50	0.18	0.52
Skewness		−4.68	−0.02	−0.5	0.54	0.05	0.10	−0.08
Std. Error of Skewness		0.03	0.03	0.03	0.03	0.03	0.03	0.03
Kurtosis		19.93	−2.000	−0.136	0.641	0.64	−0.20	−1.22
Std. Error of Kurtosis		0.06	0.06	0.06	0.06	0.06	0.06	0.06
Minimum		0.00	0.00	20.00	37.00	140.00	1.303	0.00
Maximum		1.00	1.00	88.00	134.00	184.00	2.365	2.00

**Table 3:** Histopathological and immunohistochemical characteristics of infiltrative ductal carcinoma.

Tumor Type	Grade Differentiation	Nodal Invasion	IHC Profile	FISH/CISH	Metastasis
Infiltrative ductal carcinoma	-	-	ER: 95%, PR: 98%, Ki67: 30%, HER2: 3+	-	-
Infiltrative ductal carcinoma	-	6/12	ER: 20%, PR: 90%, Ki67: 30%, HER2: 2+	CISH (+)	-
Invasive ductal carcinoma	G3	3/13	ER: 0%, PR: 0%, Ki67: 90%, HER2: 3+	-	Lymphnodular metastases
Invasive ductal carcinoma	G2	2/17	ER: 90%, PR: 0%, Ki67: 10%, HER2: 2+	CISH (+)	-
Invasive ductal and lobular carcinoma	-	-	ER: 90%, PR: 90%, Ki67: 30%, HER2: 2+	-	-
Infiltrative ductal carcinoma	-	-	ER: 0%, PR: 0%, Ki67: Neprecizat, HER2: 2+	CISH (+)	Carcinomatous emboli
Poorly differentiated ductal carcinoma	-	-	ER: 90%, PR: -, Ki67: 80%, HER2: -	-	-
Invasive ductal carcinoma	G3	1/9	ER: 60%, PR: 75%, Ki67: 30%, HER2: 2+	CISH (+)	-

IDC = Infiltrative ductal carcinoma, G2, G3 = Tumor Grade, IHC = Immunohistochemistry, ER = Estrogen Receptor, PR = Progesterone Receptor, Ki67 = Marker of cell proliferation, HER2 = Human Epidermal Growth Factor Receptor 2, FISH = Fluorescence In Situ Hybridization, CISH = Chromogenic In Situ Hybridization.

**Table 4:** TNM classification of breast cancer.

T (Tumor)	N (Nodes)	M (Metastasis)	Additional Details
T1b	N1	M0	Small tumor, 1 lymph node, no metastases
T2	N0	M1 (pul)	Moderate tumor, no lymph node involvement, lung metastases
T3	N2	M1 (oss, pul, lym)	Large tumor, extensive nodal involvement, bone, lung, and lymph node metastases
T4b	N1	M1 (pul)	Tumor with significant local extension, involvement of 1 ganglion, lung metastases
T4c	N2	M1 (oss, hep, lym)	Tumor with extensive local extension, extensive nodal involvement, bone, liver, and lymph node metastases
T4d	N3	M1 (oss, hep, pul, lym)	Tumor with extreme local extension, very extensive nodal involvement, multiple metastases in bones, liver, lungs, and lymph nodes

**Table 5:** Correlation matrix of health and performance variables.

Correlations		LVEF	Heart_Problems	Age	Weight	Height	Body Surface	Performance Index
LVEF	r	1	−0.153 **	−0.007	−0.359 **	−0.078 **	−0.308 **	−0.009
	*p*	-	0.000	0.604	0.000	0.000	0.000	0.534
Heart_problems	r	−0.153 **	1	0.138 **	0.597 **	0.203 **	0.562 **	0.096 **
	*p*	0.000	-	0.000	0.000	0.000	0.000	0.000
Age	r	−0.007	0.138 **	1	0.140 **	−0.205 **	0.052 **	0.247 **
	*p*	0.604	0.000	-	0.000	0.000	0.000	0.000
Weight	r	−0.359 **	0.597 **	0.140 **	1	0.355 **	0.938 **	0.069 **
	*p*	0.000	0.000	0.000	-	0.000	0.000	0.000
Height	r	−0.078 **	0.203 **	−0.205 **	0.355 **	1	0.632 **	−0.069 **
	*p*	0.000	0.000	0.000	0.000	-	0.000	0.000
Body_surface	r	−0.308 **	0.562 **	0.052 **	0.938 **	0.632 **	1	0.032 *
	*p*	0.000	0.000	0.000	0.000	0.000	-	0.022
Performance_index	r	−0.009	0.096 **	0.247 **	0.069 **	−0.069 **	0.032 *	1
	*p*	0.534	0.000	0.000	0.000	0.000	0.022	-
	N	5149						

r = Pearson coefficient, *p* = statistically significant, ** = correlation is significant at the 0.01 level (2-tailed). * = Correlation is significant at the 0.05 level (2-tailed).

## Data Availability

All the data processed in this article are part of the research for a doctoral thesis, being archived in the aesthetic medical office, where the interventions were performed.
